# Bis[2-(2-pyridylmethyl­eneamino)benzene­sulfonato-κ^3^ *N*,*N*′,*O*]cadmium(II) dihydrate

**DOI:** 10.1107/S1600536808034387

**Published:** 2008-10-25

**Authors:** Miao Ou-Yang, Xue-Ren Huang, Yun-Liang Zhang, Yi-Min Jiang

**Affiliations:** a College of Chemistry and Chemical Engineering, Guangxi Normal University, Guilin, Guangxi 541004, People’s Republic of China; b School of Medicine, Shao Yang Medical College, Shaoyang 422000, People’s Republic of China

## Abstract

The title complex, [Cd(Paba)_2_]·2H_2_O or [Cd(C_12_H_9_N_2_O_3_S)_2_]·2H_2_O, was synthesized by the reaction of the potassium salt of 2-(2-pyridylmethyl­eneamino)benzene­sulfonic acid (PabaK) with CdCl_2_·2.5H_2_O in methanol. The Cd^II^ atom lies on a crystallographic twofold axis and is coordinated by four N atoms and two O atoms from two deprotonated tridentate 2-(2-pyridylmethyl­eneamino)benzene­sulfonate ligands in a slightly distorted octa­hedral environment. There are extensive hydrogen bonds of the type O—H⋯O between the uncoordinated water molecules and the sulfonate O atoms, through which the complex forms a layered structure parallel to (001).

## Related literature

For the isostructural Zn compound, see: Cai *et al.* (2008 ▶). For synthesis of the ligand, see: Casella & Gullotti (1986 ▶).
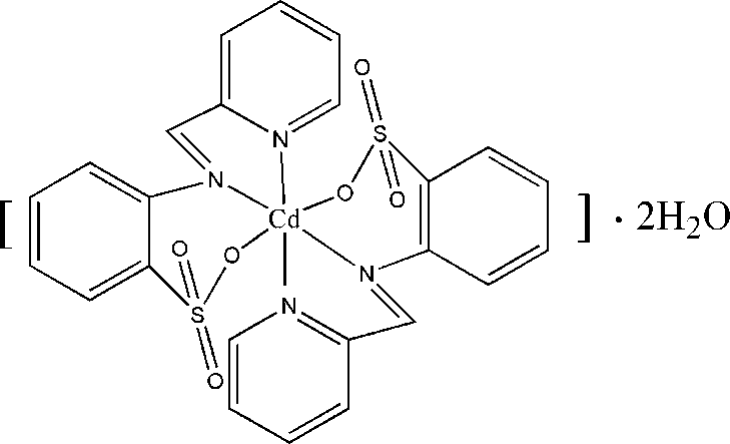


## Experimental

### 

#### Crystal data

[Cd(C_12_H_9_N_2_O_3_S)_2_]·2H_2_O
*M* *_r_* = 670.98
Orthorhombic, 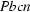

*a* = 20.255 (4) Å
*b* = 7.8924 (17) Å
*c* = 16.475 (3) Å
*V* = 2633.7 (9) Å^3^
*Z* = 4
Mo *K*α radiation
μ = 1.04 mm^−1^
*T* = 291 (2) K
0.23 × 0.08 × 0.05 mm

#### Data collection

Bruker SMART CCD area-detector diffractometer
Absorption correction: multi-scan (*SADABS*; Sheldrick, 1996 ▶) *T*_min_ = 0.798, *T*_max_ = 0.950
18106 measured reflections
2443 independent reflections
1672 reflections with *I* > 2σ(*I*)
*R*_int_ = 0.075

#### Refinement

*R*[*F* ^2^ > 2σ(*F* ^2^)] = 0.034
*wR*(*F* ^2^) = 0.075
*S* = 1.04
2443 reflections
177 parameters
H-atom parameters constrained
Δρ_max_ = 0.33 e Å^−3^
Δρ_min_ = −0.33 e Å^−3^

### 

Data collection: *SMART* (Bruker, 2004 ▶); cell refinement: *SAINT* (Bruker, 2004 ▶); data reduction: *SAINT*; program(s) used to solve structure: *SHELXS97* (Sheldrick, 2008 ▶); program(s) used to refine structure: *SHELXL97* (Sheldrick, 2008 ▶); molecular graphics: *SHELXTL* (Sheldrick, 2008 ▶); software used to prepare material for publication: *SHELXTL*.

## Supplementary Material

Crystal structure: contains datablocks I, global. DOI: 10.1107/S1600536808034387/pk2123sup1.cif

Structure factors: contains datablocks I. DOI: 10.1107/S1600536808034387/pk2123Isup2.hkl

Additional supplementary materials:  crystallographic information; 3D view; checkCIF report

## Figures and Tables

**Table 1:** Hydrogen-bond geometry (Å, °)

*D*—H⋯*A*	*D*—H	H⋯*A*	*D*⋯*A*	*D*—H⋯*A*
O4—H2*W*⋯O3^i^	0.83	2.27	2.968 (5)	142
O4—H1*W*⋯O1	0.85	2.01	2.863 (5)	179

Symmetry code: (i) 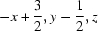.

**Fig. 1.**
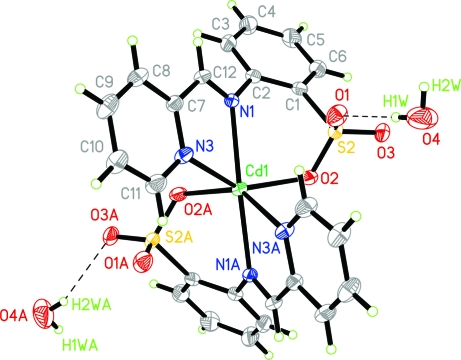
An ellipsoid plot (30% probability) showing the numbering scheme. Dashed lines indicate hydrogen bonds. Symmetry code: 1# -x + 1, y, -z + 3/2.

**Fig. 2.**
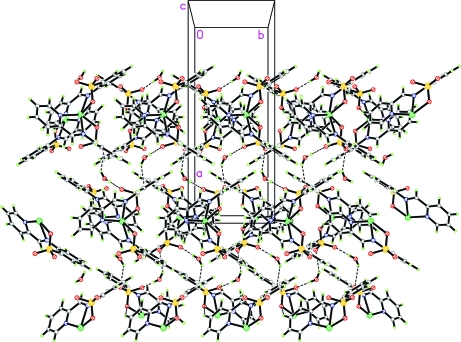
2-D network, as viewed down the c axis. Dashed lines indicate hydrogen bonds.

## supplementary crystallographic information

### Comment

The title complex (Fig. 1) is isostructural with [Zn(Paba)_2_].2H_2_O, whose structure has been described in detail (Cai, *et al.*, 2008). The six-coordinated Cd^II^ lies on a crystallographic 2-fold axis of rotation and two deprotonated PabaH anions coordinate to Cd^II^ in a facial arrangement as N,*N*',*O*-tridentate donor ligands.

The O—H donor group of the guest waters and the S=O acceptor group of the Paba ligands participate in the hydrogen bonding and form a two-dimensional network in the *ab* plane (Fig. 2).

### Experimental

The potassium salt of 2-(2- pyridylmethyleneamino)benzenesulfonic acid(PabaK) was synthesized according to the literature methods (Casella & Gullotti, 1986).

For the preparation of the title complex, the ligand PabaK (1 mmol, 0.30 g) was dissolved in methanol (10 ml) at 333 K and an aqueous solution (10 ml) containing CdCl_2_.2.5H_2_O (0.5 mmol, 0.12 g) was added. The resulting mixture was stirred at 333 K for 4 h. Then the mixture was filtrated and the filtrate was left to stand at room temperature. Yellow crystals suitable for X-ray diffraction were obtained after a week in a yield of 35%. Elemental analysis,.found (%): C, 46.91; H, 3.36; N, 8.30; S, 9.45; calc (%): C, 42.96; H, 3.31; N, 8.35; S, 9.56.

### Refinement

H atoms bonded to C were positioned geometrically with C—H distance 0.93 Å, and treated as riding atoms,with *U*_iso_(H)= 1.2*U*_eq_(*C*). Water hydrogens were placed in fixed positions and assigned U_iso _values of 1.5 U_eq _of the water oxygen atom.

### Crystal data

**Table:**

[Cd(C_12_H_9_N_2_O_3_S)_2_]·2H_2_O	*F*(000) = 1352
*M**_r_* = 670.98	*D*_x_ = 1.692 Mg m^−^^3^
Orthorhombic, *P**b**c**n*	Mo *K*α radiation, λ = 0.71073 Å
Hall symbol: -P 2n 2ab	Cell parameters from 2541 reflections
*a* = 20.255 (4) Å	θ = 2.5–23.2°
*b* = 7.8924 (17) Å	µ = 1.04 mm^−^^1^
*c* = 16.475 (3) Å	*T* = 291 K
*V* = 2633.7 (9) Å^3^	Block, colourless
*Z* = 4	0.23 × 0.08 × 0.05 mm

### Data collection

**Table:**

Bruker SMART CCD area-detector diffractometer	2443 independent reflections
Radiation source: fine-focus sealed tube	1672 reflections with *I* > 2σ(*I*)
graphite	*R*_int_ = 0.075
φ and ω scans	θ_max_ = 25.5°, θ_min_ = 2.5°
Absorption correction: multi-scan (SADABS; Sheldrick, 1996)	*h* = −24→24
*T*_min_ = 0.798, *T*_max_ = 0.950	*k* = −9→9
18106 measured reflections	*l* = −19→19

### Refinement

**Table:**

Refinement on *F*^2^	Primary atom site location: structure-invariant direct methods
Least-squares matrix: full	Secondary atom site location: difference Fourier map
*R*[*F*^2^ > 2σ(*F*^2^)] = 0.034	Hydrogen site location: inferred from neighbouring sites
*wR*(*F*^2^) = 0.075	H-atom parameters constrained
*S* = 1.04	*w* = 1/[σ^2^(*F*_o_^2^) + (0.0212*P*)^2^ + 3.1957*P*] where *P* = (*F*_o_^2^ + 2*F*_c_^2^)/3
2443 reflections	(Δ/σ)_max_ = 0.001
177 parameters	Δρ_max_ = 0.33 e Å^−^^3^
0 restraints	Δρ_min_ = −0.33 e Å^−^^3^

### Special details

**Table:**

Geometry. All e.s.d.'s (except the e.s.d. in the dihedral angle between two l.s. planes)are estimated using the full covariance matrix. The cell e.s.d.'s are takeninto account individually in the estimation of e.s.d.'s in distances, anglesand torsion angles; correlations between e.s.d.'s in cell parameters are onlyused when they are defined by crystal symmetry. An approximate (isotropic)treatment of cell e.s.d.'s is used for estimating e.s.d.'s involving l.s. planes.
Refinement. Refinement of *F*^2^ against ALL reflections. The weighted *R*-factor *wR* andgoodness of fit *S* are based on *F*^2^, conventional *R*-factors *R* are basedon *F*, with *F* set to zero for negative *F*^2^. The threshold expression of*F*^2^ > σ(*F*^2^) is used only for calculating *R*-factors(gt) *etc*. and isnot relevant to the choice of reflections for refinement. *R*-factors basedon *F*^2^ are statistically about twice as large as those based on *F*, and *R*-factors based on ALL data will be even larger.

### Fractional atomic coordinates and isotropic or equivalent isotropic displacement parameters (Å^2^)

**Table:**

	*x*	*y*	*z*	*U*_iso_*/*U*_eq_	
Cd1	0.5000	0.68040 (5)	0.7500	0.03074 (13)	
S2	0.62752 (5)	0.83468 (15)	0.66388 (6)	0.0372 (3)	
N1	0.59409 (15)	0.7358 (4)	0.83494 (18)	0.0302 (7)	
C12	0.60345 (19)	0.6263 (5)	0.8904 (2)	0.0382 (10)	
H12	0.6393	0.6381	0.9253	0.046*	
N3	0.50869 (16)	0.4691 (4)	0.84673 (18)	0.0373 (8)	
O1	0.64757 (15)	0.6598 (4)	0.67446 (17)	0.0523 (8)	
O2	0.55529 (12)	0.8506 (4)	0.66248 (15)	0.0413 (7)	
O3	0.65696 (14)	0.9203 (4)	0.59556 (16)	0.0509 (8)	
O4	0.69916 (18)	0.4179 (6)	0.5615 (2)	0.1121 (17)	
H1W	0.6840	0.4904	0.5948	0.168*	
H2W	0.7324	0.3778	0.5833	0.168*	
C1	0.65210 (16)	0.9451 (5)	0.7530 (3)	0.0342 (9)	
C2	0.63521 (18)	0.8827 (5)	0.8292 (2)	0.0319 (9)	
C3	0.6563 (2)	0.9697 (6)	0.8973 (3)	0.0455 (11)	
H3	0.6462	0.9281	0.9486	0.055*	
C4	0.6920 (2)	1.1170 (6)	0.8899 (3)	0.0552 (13)	
H4	0.7057	1.1748	0.9361	0.066*	
C5	0.7074 (2)	1.1785 (6)	0.8147 (3)	0.0561 (13)	
H5	0.7310	1.2790	0.8099	0.067*	
C6	0.6880 (2)	1.0922 (5)	0.7457 (3)	0.0481 (11)	
H6	0.6992	1.1333	0.6947	0.058*	
C7	0.55885 (19)	0.4823 (5)	0.9002 (2)	0.0334 (9)	
C8	0.5667 (2)	0.3684 (5)	0.9628 (2)	0.0445 (11)	
H8	0.6020	0.3780	0.9985	0.053*	
C9	0.5212 (2)	0.2402 (6)	0.9716 (3)	0.0520 (13)	
H9	0.5253	0.1625	1.0138	0.062*	
C10	0.4700 (2)	0.2279 (5)	0.9178 (3)	0.0491 (12)	
H10	0.4387	0.1424	0.9232	0.059*	
C11	0.4654 (2)	0.3423 (6)	0.8565 (3)	0.0465 (11)	
H11	0.4308	0.3322	0.8197	0.056*	

### Atomic displacement parameters (Å^2^)

**Table:**

	*U*^11^	*U*^22^	*U*^33^	*U*^12^	*U*^13^	*U*^23^
Cd1	0.0291 (2)	0.0362 (2)	0.0269 (2)	0.000	−0.00588 (19)	0.000
S2	0.0355 (6)	0.0497 (7)	0.0265 (5)	−0.0025 (6)	−0.0008 (4)	0.0002 (5)
N1	0.0246 (16)	0.0403 (19)	0.0257 (17)	−0.0062 (15)	−0.0016 (14)	0.0016 (16)
C12	0.032 (2)	0.055 (3)	0.027 (2)	0.000 (2)	−0.0072 (18)	−0.001 (2)
N3	0.043 (2)	0.0398 (19)	0.0289 (17)	−0.0012 (18)	−0.0090 (17)	0.0029 (15)
O1	0.065 (2)	0.047 (2)	0.0442 (18)	0.0116 (16)	−0.0022 (15)	−0.0107 (15)
O2	0.0329 (15)	0.059 (2)	0.0322 (15)	−0.0061 (14)	−0.0076 (12)	0.0112 (14)
O3	0.0464 (18)	0.077 (2)	0.0296 (16)	−0.0113 (17)	0.0048 (14)	0.0037 (16)
O4	0.066 (2)	0.168 (4)	0.102 (3)	0.043 (3)	−0.025 (2)	−0.068 (3)
C1	0.0245 (18)	0.044 (2)	0.034 (2)	0.0008 (16)	−0.004 (2)	−0.004 (2)
C2	0.024 (2)	0.037 (2)	0.034 (2)	0.0001 (18)	−0.0021 (18)	−0.0005 (19)
C3	0.042 (3)	0.062 (3)	0.032 (2)	−0.007 (2)	0.001 (2)	−0.004 (2)
C4	0.053 (3)	0.070 (3)	0.043 (3)	−0.020 (3)	−0.002 (2)	−0.019 (2)
C5	0.048 (3)	0.058 (3)	0.062 (3)	−0.026 (3)	0.002 (2)	−0.011 (3)
C6	0.042 (2)	0.057 (3)	0.045 (3)	−0.012 (2)	0.001 (2)	0.000 (3)
C7	0.035 (2)	0.036 (2)	0.029 (2)	−0.0003 (19)	−0.0025 (18)	−0.0014 (18)
C8	0.052 (3)	0.047 (3)	0.035 (2)	0.006 (2)	−0.011 (2)	0.005 (2)
C9	0.081 (4)	0.037 (3)	0.038 (3)	0.002 (2)	−0.007 (2)	0.010 (2)
C10	0.071 (3)	0.036 (3)	0.040 (3)	−0.013 (2)	−0.005 (2)	0.001 (2)
C11	0.055 (3)	0.045 (3)	0.039 (3)	−0.013 (2)	−0.010 (2)	0.004 (2)

### Geometric parameters (Å, °)

**Table:**

Cd1—O2^i^	2.267 (3)	C1—C6	1.375 (5)
Cd1—O2	2.267 (3)	C1—C2	1.390 (5)
Cd1—N3^i^	2.313 (3)	C2—C3	1.383 (5)
Cd1—N3	2.313 (3)	C3—C4	1.374 (6)
Cd1—N1^i^	2.405 (3)	C3—H3	0.9300
Cd1—N1	2.405 (3)	C4—C5	1.367 (6)
S2—O3	1.442 (3)	C4—H4	0.9300
S2—O1	1.449 (3)	C5—C6	1.381 (6)
S2—O2	1.469 (3)	C5—H5	0.9300
S2—C1	1.779 (4)	C6—H6	0.9300
N1—C12	1.272 (5)	C7—C8	1.377 (5)
N1—C2	1.431 (5)	C8—C9	1.376 (6)
C12—C7	1.461 (5)	C8—H8	0.9300
C12—H12	0.9300	C9—C10	1.368 (6)
N3—C11	1.340 (5)	C9—H9	0.9300
N3—C7	1.349 (5)	C10—C11	1.359 (6)
O4—H1W	0.8502	C10—H10	0.9300
O4—H2W	0.8263	C11—H11	0.9300
			
O2^i^—Cd1—O2	107.31 (15)	C6—C1—S2	119.3 (4)
O2^i^—Cd1—N3^i^	145.88 (11)	C2—C1—S2	120.2 (3)
O2—Cd1—N3^i^	91.52 (11)	C3—C2—C1	118.7 (4)
O2^i^—Cd1—N3	91.52 (11)	C3—C2—N1	121.9 (4)
O2—Cd1—N3	145.88 (11)	C1—C2—N1	119.3 (3)
N3^i^—Cd1—N3	87.74 (16)	C4—C3—C2	120.7 (4)
O2^i^—Cd1—N1^i^	82.58 (10)	C4—C3—H3	119.6
O2—Cd1—N1^i^	85.04 (10)	C2—C3—H3	119.6
N3^i^—Cd1—N1^i^	70.73 (11)	C5—C4—C3	120.0 (4)
N3—Cd1—N1^i^	126.32 (11)	C5—C4—H4	120.0
O2^i^—Cd1—N1	85.04 (10)	C3—C4—H4	120.0
O2—Cd1—N1	82.58 (10)	C4—C5—C6	120.4 (4)
N3^i^—Cd1—N1	126.32 (11)	C4—C5—H5	119.8
N3—Cd1—N1	70.73 (11)	C6—C5—H5	119.8
N1^i^—Cd1—N1	159.04 (16)	C1—C6—C5	119.6 (5)
O3—S2—O1	115.11 (19)	C1—C6—H6	120.2
O3—S2—O2	111.07 (17)	C5—C6—H6	120.2
O1—S2—O2	111.26 (18)	N3—C7—C8	121.7 (4)
O3—S2—C1	107.40 (18)	N3—C7—C12	117.0 (4)
O1—S2—C1	106.80 (18)	C8—C7—C12	121.3 (4)
O2—S2—C1	104.47 (17)	C9—C8—C7	118.8 (4)
C12—N1—C2	120.8 (3)	C9—C8—H8	120.6
C12—N1—Cd1	114.4 (3)	C7—C8—H8	120.6
C2—N1—Cd1	124.8 (2)	C10—C9—C8	119.4 (4)
N1—C12—C7	121.1 (4)	C10—C9—H9	120.3
N1—C12—H12	119.5	C8—C9—H9	120.3
C7—C12—H12	119.5	C11—C10—C9	119.2 (4)
C11—N3—C7	118.2 (3)	C11—C10—H10	120.4
C11—N3—Cd1	124.8 (3)	C9—C10—H10	120.4
C7—N3—Cd1	116.9 (3)	N3—C11—C10	122.7 (4)
S2—O2—Cd1	115.56 (15)	N3—C11—H11	118.6
H1W—O4—H2W	105.8	C10—C11—H11	118.6
C6—C1—C2	120.5 (4)		
			
O2^i^—Cd1—N1—C12	93.5 (3)	O2—S2—C1—C6	−113.1 (3)
O2—Cd1—N1—C12	−158.3 (3)	O3—S2—C1—C2	−174.9 (3)
N3^i^—Cd1—N1—C12	−71.9 (3)	O1—S2—C1—C2	−50.9 (3)
N3—Cd1—N1—C12	0.1 (3)	O2—S2—C1—C2	67.1 (3)
N1^i^—Cd1—N1—C12	147.4 (3)	C6—C1—C2—C3	−1.2 (6)
O2^i^—Cd1—N1—C2	−82.9 (3)	S2—C1—C2—C3	178.6 (3)
O2—Cd1—N1—C2	25.3 (3)	C6—C1—C2—N1	175.8 (3)
N3^i^—Cd1—N1—C2	111.7 (3)	S2—C1—C2—N1	−4.3 (5)
N3—Cd1—N1—C2	−176.3 (3)	C12—N1—C2—C3	−39.6 (5)
N1^i^—Cd1—N1—C2	−29.0 (3)	Cd1—N1—C2—C3	136.5 (3)
C2—N1—C12—C7	175.6 (3)	C12—N1—C2—C1	143.4 (4)
Cd1—N1—C12—C7	−0.9 (5)	Cd1—N1—C2—C1	−40.4 (4)
O2^i^—Cd1—N3—C11	91.9 (3)	C1—C2—C3—C4	1.4 (6)
O2—Cd1—N3—C11	−143.3 (3)	N1—C2—C3—C4	−175.5 (4)
N3^i^—Cd1—N3—C11	−53.9 (3)	C2—C3—C4—C5	−0.4 (7)
N1^i^—Cd1—N3—C11	10.0 (4)	C3—C4—C5—C6	−0.9 (7)
N1—Cd1—N3—C11	176.1 (3)	C2—C1—C6—C5	−0.1 (6)
O2^i^—Cd1—N3—C7	−83.5 (3)	S2—C1—C6—C5	−179.9 (3)
O2—Cd1—N3—C7	41.3 (4)	C4—C5—C6—C1	1.1 (7)
N3^i^—Cd1—N3—C7	130.7 (3)	C11—N3—C7—C8	0.9 (6)
N1^i^—Cd1—N3—C7	−165.4 (2)	Cd1—N3—C7—C8	176.6 (3)
N1—Cd1—N3—C7	0.7 (3)	C11—N3—C7—C12	−177.2 (4)
O3—S2—O2—Cd1	168.04 (16)	Cd1—N3—C7—C12	−1.4 (4)
O1—S2—O2—Cd1	38.4 (2)	N1—C12—C7—N3	1.6 (6)
C1—S2—O2—Cd1	−76.5 (2)	N1—C12—C7—C8	−176.5 (4)
O2^i^—Cd1—O2—S2	118.00 (19)	N3—C7—C8—C9	−1.3 (6)
N3^i^—Cd1—O2—S2	−90.86 (18)	C12—C7—C8—C9	176.7 (4)
N3—Cd1—O2—S2	−2.6 (3)	C7—C8—C9—C10	0.6 (7)
N1^i^—Cd1—O2—S2	−161.36 (19)	C8—C9—C10—C11	0.4 (7)
N1—Cd1—O2—S2	35.59 (17)	C7—N3—C11—C10	0.2 (6)
O3—S2—C1—C6	4.9 (4)	Cd1—N3—C11—C10	−175.2 (3)
O1—S2—C1—C6	128.9 (3)	C9—C10—C11—N3	−0.9 (7)

Symmetry codes: (i) −*x*+1, *y*, −*z*+3/2.

### Hydrogen-bond geometry (Å, °)

**Table:**

*D*—H···*A*	*D*—H	H···*A*	*D*···*A*	*D*—H···*A*
O4—H2W···O3^ii^	0.83	2.27	2.968 (5)	142
O4—H1W···O1	0.85	2.01	2.863 (5)	179

Symmetry codes: (ii) −*x*+3/2, *y*−1/2, *z*.

## References

[bb1] Bruker (2004). *SMART* and *SAINT* Bruker AXS inc., Madison, Wisconsin, USA.

[bb2] Cai, C.-X., Ou-Yang, M., Zhao, Z.-Y. & Jiang, Y.-M. (2008). *Acta Cryst.* E**64**, m1195. 10.1107/S1600536808026342 PMC2960510 21201634

[bb3] Casella, L. & Gullotti, M. (1986). *Inorg. Chem.* **25**, 1293–1303.

[bb4] Sheldrick, G. M. (1996). *SADABS* University of Göttingen, Germany.

[bb5] Sheldrick, G. M. (2008). *Acta Cryst.* A**64**, 112–122. 10.1107/S0108767307043930 18156677

